# Proton pump inhibitor in the prevention of upper gastrointestinal mucosal injury associated with dual antiplatelet therapy after coronary artery bypass grafting (DACAB-GI-2): study protocol for a randomized controlled trial

**DOI:** 10.1186/s13063-022-06464-w

**Published:** 2022-07-15

**Authors:** Yunpeng Zhu, Xiaojin Wang, Yi Yang, Lei Liu, Qiang Zhao, Lifen Yu

**Affiliations:** 1 Department of Cardiovascular Surgery, Ruijin Hospital, Shanghai Jiao Tong University School of Medicine, Shanghai, 200025 China; 2 Department of Gastroenterology, Ruijin Hospital, Shanghai Jiao Tong University School of Medicine, Shanghai, 200025 China

**Keywords:** Coronary artery bypass grafting, Dual antiplatelet therapy, Proton pump inhibitor, Gastrointestinal mucosal injury

## Abstract

**Background:**

Dual antiplatelet therapy (DAPT) is recommended in secondary prevention after coronary artery bypass grafting (CABG), but it is inevitably associated with the risk of bleeding, of which gastrointestinal bleeding accounts for more than half. Proton pump inhibitors (PPIs) may increase the risk of major cardiovascular adverse events when reducing the risk of upper gastrointestinal bleeding. Therefore, the optimal duration of a PPI in combination with DAPT is unclear.

**Methods:**

The “Proton Pump Inhibitor Preventing Upper Gastrointestinal Injury in Patients on Dual Antiplatelet Therapy after CABG” (DACAB-GI-2) study is a prospective, single-center, open-label, parallel, randomized controlled trial. A total of 232 eligible subjects who are scheduled or initiated on DAPT (clopidogrel plus aspirin or ticagrelor plus aspirin) for 12 months immediately after CABG will be enrolled and be randomized in a 1:1 ratio to either a 12-month pantoprazole treatment arm or a 1-month treatment arm. The primary outcome is to assess the rate of gastroduodenal erosions and ulcers evaluated by esophagogastroduodenoscopy (EGD) within 12 months after randomization, based on the modified Lanza score. Secondary outcomes include reflux esophagitis and upper gastrointestinal bleeding. Other pre-specified outcomes include major adverse cardiovascular events, graft failure, and all-cause death.

**Discussion:**

This study aims to compare the efficacy and safety of 12 months and 1 month of pantoprazole treatment in preventing DAPT-related upper gastrointestinal mucosal injury after CABG.

**Trial registration:**

ClinicalTrials.gov NCT03908593.

## Background

Dual antiplatelet therapy (DAPT) involving P2Y_12_ receptor inhibitors (clopidogrel or ticagrelor) and aspirin is recommended for secondary prevention after coronary artery bypass grafting (CABG) since 2015 [1]. However, it is associated with an increased risk of gastrointestinal bleeding events [2]. Proton pump inhibitors (PPIs) are the preferred drug for treating gastroduodenal mucosal injury and decreasing the risk of upper gastrointestinal bleeding [3–5]. Regarding the combined use with clopidogrel, previous studies have shown that omeprazole and esomeprazole have competitive inhibition on the CYP2C19 pathway [6, 7]. The guidelines recommend rabeprazole or pantoprazole in combination treatment [8, 9]. Unlike clopidogrel, ticagrelor is a non-prodrug that does not influence the five types of PPI [10, 11].

In 2018, the ESC guidelines recommended (increased from evidence IIa to Ib) a PPI in combination with DAPT treatment [8]. Our previous study (DACAB-GI-1) was a single-center randomized controlled trial (RCT) focused on upper gastrointestinal mucosal injury associated with ticagrelor plus aspirin, ticagrelor alone, or aspirin alone at 1-year post-CABG. The results showed that patients with a history of peptic ulcer and/or gastrointestinal bleeding received more attention, of which 77.7% (7/9) received PPI combination treatment for ≥6 months, while among the patients who received 1 year of ticagrelor plus aspirin therapy after CABG, only 9.9% (8/81) received PPI combination treatment for ≥6 months [12]. The upper gastrointestinal mucosal injury associated with DAPT in patients without a history of peptic ulcer and/or gastrointestinal bleeding needed to be further investigated.

Comparing with capsule endoscopy, the first advantage of esophagogastroduodenoscopy (EGD) is the capability of biopsy [13]. Overt or occult gastrointestinal bleeding in patients on DAPT not only indicates mucosal injury but also is an alarming symptom of gastrointestinal tumors [14, 15]. Pathological biopsy results are the gold standard for determining precancerous or malignant lesions of the esophagus and/or stomach, and whether *Helicobacter pylori* (*H. pylori*) infection is present. Second, EGD can clearly observe the erosion of esophageal mucosa caused by reflux esophagitis (RE), as well as the presence of cardia relaxation and hiatus hernia, which are the pathological structural basis of recurrent gastroesophageal reflux disease [16]. EGD findings could help to rule out esophageal disorders in the differential diagnosis in patients reporting retrosternal discomfort (including chest pain) after CABG. However, capsule endoscopy only remains in the esophagus for a very short time during the examination. Even magnetically controlled capsule endoscopy cannot achieve a subtle examination of several sites, such as the esophagus and cardia [17]. Third, the total medical cost for anesthesia EGD (including pathological biopsy examination) in China is far lower than that of magnetically controlled capsule endoscopy (less than 1/3 of the total cost of the latter) and is included in the national medical insurance coverage.

To date, there has been no study using EGD to compare the differences in upper gastrointestinal mucosal injury after 6 and 12 months of PPI treatment combined with two different DAPT regimens (clopidogrel plus aspirin or ticagrelor plus aspirin). The present study (DACAB-GI-2) intends to compare the efficacy and safety of continuous treatment with pantoprazole for 12 vs. 1 month in preventing DAPT-related upper gastrointestinal mucosal injury after CABG.

## Methods

### Study design

This prospective trial was designed as a single-center randomized, open-label, parallel controlled trial with patients allocated in a ratio of 1:1, to be conducted at the Ruijin Hospital, Shanghai Jiao Tong University School of Medicine, Shanghai, China. The protocol was designed according to the Standard Protocol Items: Recommendations for Interventional Trials (SPIRIT) 2013 Statement.

### Study objectives and hypothesis

The primary objective of the DACAB-GI-2 study is to assess the effect of pantoprazole in the prevention of upper gastrointestinal mucosal injury in patients on DAPT after CABG. The secondary objective is to determine the cumulative incidence of upper gastrointestinal bleeding and major adverse cardiovascular events (MACE). We predicted that patients treated with pantoprazole for 12 months would have a significantly less upper gastrointestinal mucosal injury than patients treated with pantoprazole for 1 month.

### Ethics issues

The present study protocol was approved by the Ethics Committee of Ruijin Hospital, Shanghai Jiao Tong University School of Medicine (No. 2019(43)).

### Participant recruitment

This clinical study was registered on October 20, 2019. Participants will be screened from patients who have undergone CABG at the Cardiac Surgery Department, Ruijin Hospital, after registration. Within 3 days after surgery, patients willing to participate in the study will be carefully evaluated by a cardiac surgeon together with a gastroenterologist based on inclusion and exclusion criteria.

### Criteria

#### Inclusion criteria

1. Provision of signed informed consent before any study-specific procedures
2. Male or female, age ≥ 18 years at the time of consent
3. Planned or initiated the use of 12 months of DAPT (clopidogrel plus aspirin or ticagrelor plus aspirin) immediately following primary isolated elective CABG surgery

#### Exclusion criteria

1. History of the previous active peptic ulcer within 3 months before enrollment
2. Planned use of PPIs to treat acid-associated disorders (e.g., gastroesophageal reflux disease, GERD)
3. Contraindications for aspirin, clopidogrel, ticagrelor, and pantoprazole use (e.g., known allergy)
4. Anticipated concomitant oral or intravenous therapy with strong cytochrome P450 3A4 (CYP3A4) inhibitors or CYP3A4 substrates with narrow therapeutic indices that cannot be stopped for the course of the study
  1. Strong inhibitors: ketoconazole, itraconazole, voriconazole, telithromycin, clarithromycin, nefazodone, ritonavir, saquinavir, nelfinavir, indinavir, and atazanavir
  2. CYP3A4 substrates with narrow therapeutic index: quinidine, simvastatin at doses > 40mg daily or lovastatin at doses > 40mg daily
5. Need for chronic oral anticoagulant therapy or chronic low-molecular-weight heparin
6. Women of child-bearing potential who are not willing to use a medically accepted method of contraception that is considered reliable in the judgment of the investigator or women who have a positive pregnancy test at enrollment or randomization or women who are breasting feeding
7. Inability of the patient to understand and/or comply with study procedures and/or follow-up, in the opinion of the investigator, or any conditions that, in the opinion of the investigator, may render the patient unable to complete the study
8. Any condition outside the atherothrombotic study area with a life expectancy of less than 1 year
9. Participation in another clinical study with an investigational product within 28 days before enrollment or previous randomization to an investigational product in another ongoing clinical study
10. Any condition which in the opinion of the investigator would make it unsafe or unsuitable for the patient to participate in this study (e.g., long-term concomitant treatment with non-steroidal anti-inflammatory drugs [NSAIDs])

### Informed consent

Before enrollment, trained researchers will introduce the objective and main aspects of the trial to the patients. Patients will also receive information sheets and then be able to have an informed discussion with the participating consultant. Patients will also be informed of the probable benefits and potential risks and assured that participation is entirely voluntary. Patients who meet all of the inclusion criteria and none of the exclusion criteria will be enrolled after providing written informed consent. The personal information of all participants will always be kept confidential.

### Randomization and allocation

**Fig. 1:**
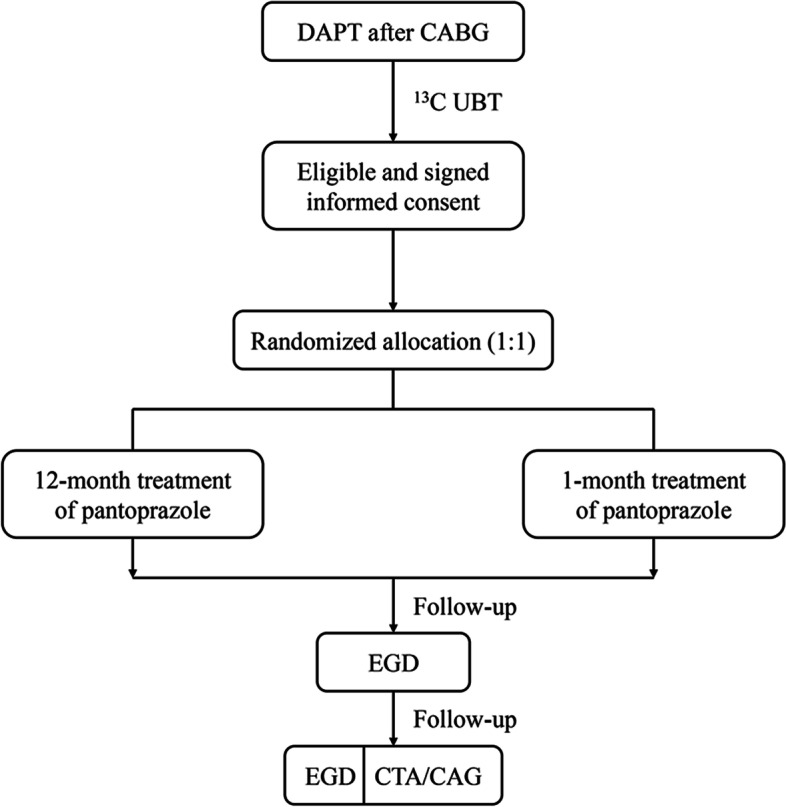
Trial flow diagram

The eligible subjects will be randomized in a ratio of 1:1 to the 12-month or the 1-month pantoprazole treatment group. Block randomization with a block length of four will be applied. The randomization numbers will be generated in a list using SAS 9.4 (SAS Institute Inc., Cary, NC, USA) and sealed in the envelopes (Fig. 1).

### Blinding

This is an open-label trial.

### Intervention

**Fig. 2:**
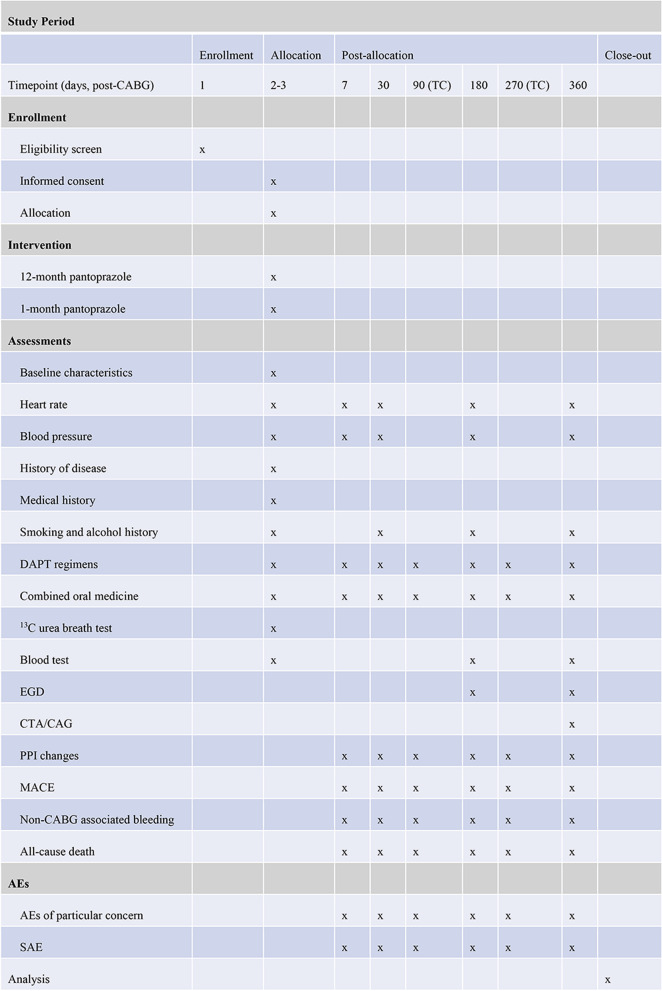
Schedule of enrollment, interventions, and assessments for the trial

Participants will be equally randomized into two groups: the experimental group (12-month treatment of pantoprazole) or the control group (1-month treatment of pantoprazole). Pantoprazole, 40 mg daily, will be used in combination with two different DAPT regimens (clopidogrel plus aspirin or ticagrelor plus aspirin), respectively. The study drug will be provided by Takeda (Shanghai, China). The detailed study schedule is listed in Fig. 2.

Relevant concomitant interventions that are permitted or prohibited during the trial: The medication for secondary prevention after CABG may follow the guidelines for cardiovascular disease. The use of anti-dyslipidemic, anti-hypertensive, and anti-diabetic medications is permitted. If the following two situations occur, it will not be regarded as a violation of the study protocol: (1) The cumulative number of days that a patient has taken double doses of PPI should not exceed 1 month. (2) The cumulative number of days that a patient has taken NSAIDs should not exceed 2 months. In the present study, patients randomized in the 1-month pantoprazole treatment group are allowed to take histamine-2 receptor blocker or gastric mucosal protective agents as needed after withdrawal of pantoprazole. However, patients randomized in the 12-month pantoprazole treatment group are prohibited to use the above medication. If a patient receives *H. pylori* eradication therapy and the second urea breath test within the 12 months after randomization, he should report to the investigator.

### Data capture

The case report form (CRF) will be finalized and the electronic database will be established based on CRF before the enrollment of the first subject. All required study data must be recorded in the CRFs. The training on CRF implementation will be provided to all the staff on the site, including instructions on how to address missing data, corrections, query procedures, and signatures. For those subjects who withdraw before completion of the study, all available efficacy and safety data must be recorded in the CRFs. Incomplete or inconsistent data on the CRFs will result in data queries addressed to the investigator for resolution.

### Follow-up

Patients will be followed up to 12 months post-randomization unless dead. The outcome will be collected at 2±1 day, 9±2 days, 1 month (30±7 days), 3 months (90±7 days), 6 months (180±14 days), 9 months (270±14 days), and 12 months (360±14 days) after CABG surgery, respectively. Among them, the visits at 3 and 9 months after randomization are telephone calls. Each subject’s survival status (surviving/deceased), blood pressure, heart rate, body mass index, waist circumference, and adverse events (AEs) will be recorded on the CRF after each conversation. At 6 and 12 months post-randomization, routine laboratory tests and anesthesia EGD will be performed respectively. The endoscopist in the present study who evaluated the upper GI mucosal injury was blinded to random treatment allocations. In addition, computed tomographic angiography (CTA) or coronary angiography (CAG) is also required at 12 months post-randomization. Face-to-face adherence reminder sessions will take place at the initial pantoprazole dispensing and each study visit thereafter.

### Withdrawal

Subjects may voluntarily withdraw from the study at any time without having to provide a reason. If the subject’s compliance is poor, the principal investigator reserves the final right to remove it from this study.

Subjects may be withdrawn because of the following reasons:

1. Major protocol deviations
2. Due to safety concerns, patients with AEs are not suitable for continued participation in this study
3. Other situations: based on the investigator’s discretion, the patient is no longer eligible for the study for any reason
4. Missed follow-up visit or death

### Sample size

According to the results of the preliminary study, the cumulative incidence of severe gastric and duodenal bulb erosion and ulcers of the 1-month PPI treatment group is assumed to be 36%, and that of the 12-month PPI treatment group was assumed to be 18%. Based on a two-sided *α* of 0.05 and a randomization ratio of 1:1, a total of 186 subjects are required to provide a power of 80%. Considering a 20% drop-out rate, the sample size of 232 subjects was determined.

### Scope of study data

At the first visit, we will collect the demographic information of the participants, including age, gender, past medication history, medical history, smoking, and alcohol use history. The results of hemoglobin, platelet count, liver function, renal function, serum lipid analysis, HbA1c, and DIC tested for the first time after admission are used as baseline data. The above laboratory tests will be also collected and recorded at 6 and 12 months after randomization. ^13^C urea breath test should be done before enrollment.

### Outcome measurements

The primary outcome of this study will be the percentage of patients with gastroduodenal erosions and ulcers evaluated by EGD within 12 months after randomization. Gastroduodenal erosions and ulcers will be assessed according to the Lanza Endoscopic Scoring System [18] (0, normal; 1, mucosal hemorrhages only; 2, 1–2 erosions; 3, 3–10 erosions; 4, >10 erosions or an ulcer ≥3 mm). In addition, ≥1 ulcer with a diameter no less than 5mm will be grouped separately.

Secondary outcomes are as follows:

1. Percentage of patients with gastroduodenal erosions and ulcers evaluated by EGD at 6 months after randomization.
2. Cumulative rate of patients with upper gastrointestinal bleeding according to the modified TIMI criteria. According to the definition of non-CABG-related bleeding in the modified TIMI criteria, the severity of upper gastrointestinal bleeding is classified into 3 grades: major, minor, and minimal—(1) major: clinically overt signs of hemorrhage associated with a drop in hemoglobin of ≥5 g/dL; (2) minor: clinically overt signs of upper gastrointestinal bleeding, resulting in hemoglobin drop of 3 to <5 g/dL; and (3) minimal: any overt bleeding event that does not meet the criteria above.
3. Percentage of patients with RE evaluated by EGD at 12 months after randomization. According to the Los Angeles classification, endoscopic reflux esophagitis was assigned a grade from A to D.
4. Percentage of patients with RE evaluated by EGD at 6 months after randomization.

Other pre-specified outcomes (up to 12 months) include:

1. The cumulative rate of patients with MACE (composite of cardiovascular death, myocardial infarctions, or stroke)
2. The failure rate of grafts assessed by computed tomographic angiography or coronary angiography. Graft failure was assessed according to the modified Fitzgibbon classification [19]: stenosis ≥50% or occlusion of the graft or distal anastomosis (type B or O)
3. The cumulative rate of patients with all-cause death

### Definition of the end of study

1. The date on which the last subject completed the last follow-up was the endpoint of this study
2. During the follow-up period, if the patient’s biopsy pathological report showed that EGD detects precancerous lesions (e.g., atypical hyperplasia, low-grade or high-grade internal neoplasia, etc.) or malignant lesions
3. Patients randomized to the control group (1-month pantoprazole treatment group) should continue PPI treatment if GERD-Q questionnaire score ≥ 8, endoscopic findings of reflux esophagitis (LA-A and above), gastroduodenal mucosal injury of modified Lanza score of 4, or upper gastrointestinal bleeding at any time during the follow-up period

According to the ITT principle, all patients randomized in this study should be evaluated for primary and secondary endpoints regardless of changes in PPI treatment. Thus, patients in the 1-month pantoprazole treatment group required PPI treatment at any time after 1 month and could also be assessed for primary and secondary endpoints.

### Statistical and analytical plans

The primary analysis was conducted according to the intention-to-treat (ITT) principle. All randomized subjects were included in the ITT analysis set. Subjects who received at least one dose of study medication and continued to receive the randomized intervention regimen (with or without study medication) 1 month after CABG were defined as the modified full analysis set (mFAS) population. Among them, subjects who completed the treatment according to the randomized protocol without major protocol violations and had a primary outcome assessment were included in the per-protocol set. Effectiveness analysis will be performed on the basis of mFAS. The safety analysis set consists of all the subjects who had taken at least one dose of study medication.

In this study, all subjects received pantoprazole treatment for at least 1 month after CABG. Due to the COVID-19 pandemic, some subjects are unable to return to the center for study drugs and/or follow-up 1 month after the randomization. Those subjects who are unable to receive subsequent randomization intervention will be excluded from the mFAS and not included in the effectiveness analysis. Considering the statistical power of the study, randomized subjects will be added until the effective randomized intervention number reaches the predetermined sample size.

All statistical analyses will be performed using SAS version 9.4 (SAS Institute Inc. SAS/STAT, Cary, NC, USA). Unless specified otherwise, the number of subjects (*n*), mean, standard deviation, median, maximum, and minimum will be provided for continuous variables, and the number of subjects and the percentage will be provided for categorical variables.

The baseline in this study will be defined as the last available (non-missing) assessment (scheduled or unscheduled) before or on the same day as the first dose of study treatment.

The chi-square test will be applied as the main analysis of the primary efficacy endpoint, and multiple sensitivity analyses will be performed to support the main analysis. For secondary and exploratory efficacy endpoints, analysis of covariance, Wilcoxon-Mann-Whitney test, log-rank test, a mixed-effects model for repeated measures, and negative binomial regression model will be applied based on the type of data in the analysis. Descriptive statistics will be provided to summarize safety endpoints.

### Monitoring

This study will be performed by the approved protocol. It is supervised by a data and safety monitoring team appointed by the ethics committee of Ruijin Hospital, Shanghai Jiao Tong University School of Medicine. The data and safety monitoring team will review the data after 20%, 50%, and 80% enrollment to monitor the study progress and all AEs that may occur. Computer-generated and timestamped audit trials will also be implemented for tracking changes in the electronic source documentation to ensure the integrity of the research data.

### Safety and adverse events

Pantoprazole tablet has been used for more than 20 years, and the overall characteristics of its adverse reactions have been well described. This study mainly collects those AEs of particular concern related to PPI [20], such as chronic nephropathy, pathological fractures, myocardial infarction, pneumonia, spontaneous bacterial peritonitis, gastrointestinal tumors, and trace element deficiency. Non-serious AEs that did not cause discontinuation of the study drug will not be collected.

Participants showing any AE will be treated appropriately by doctors, and the project will cover the cost of AEs. Adverse reactions will be checked at every visit. For any AE that occurs, all details, including the time of occurrence, symptoms, signs, degree, duration, laboratory findings, treatment, outcomes, and causal relationship with the treatment, will be recorded in the CRF. Serious AEs will be reported to the Research Ethics Committee within 24 h, which will decide whether any additional measures should be taken. The total number of patients with AEs related to the study drug (certain, probable, possible) and the total number of patients with AEs leading to discontinuation of study treatment will be summarized.

### Amendment

All proposed protocol changes will be recorded in a protocol amendment, and this will be submitted to the Committee of Ethics and the regulatory authority for approval.

## Discussion

Our previous study (DACAB-GI-1), a single-center prospective substudy of a RCT (DACAB, NCT02201771) [20], provided important reference data for the subsequent design of the DACAB-GI-2 trial. Briefly, patients aged 18–80 years with indications for elective CABG surgery were randomized to 1 year of open-label antiplatelet therapy comprising ticagrelor 90 mg twice daily plus aspirin 100 mg enteric-coated tablet, once daily; ticagrelor alone (90 mg twice daily); or aspirin alone (100 mg once daily). Prophylactic use of PPI was not a randomized intervention. It was recorded at 1, 3, 6, and 12 months post-CABG. Data of both upper gastrointestinal mucosal injury assessed by EGD and *H. pylori* infection detected by ^13^C urea breath test were obtained only at 1 year post-CABG, not at baseline [12]. In the present study (DACAB-GI-2), 232 subjects on DAPT after CABG will be randomized into two treatment groups (12- vs. 1-month treatment of pantoprazole); ^13^C urea breath test is done before enrollment and EGD is performed at 6 and 12 months after randomization, respectively.

A PPI selected in the present study was pantoprazole. Compared with the other PPIs, pantoprazole has a lower affinity for CYP2C19 and has the least interaction with other kinds of drugs, so it has higher safety and is less likely to cause AEs in patients when used in combination with dual antiplatelet agents [6, 7]. Whether the P2Y_12_ receptor inhibitor is clopidogrel or ticagrelor, pantoprazole is suitable in combination with it. In the present study, all patients will be treated with pantoprazole for at least 1 month after CABG to reduce the risk of perioperative gastrointestinal bleeding.

Patients undergoing CABG are those with more advanced coronary artery disease. EGD will not be performed preoperatively, perioperatively, and within 6 months of surgery, except in cases of emergency [21], because it is not considered to be in the patients’ best interest. In the present study, EGD will be performed at 6 and 12 months after randomization. The purpose of designing the first EGD at 6 months is not only to establish self-control but also to help identify patients with asymptomatic ulcers on time. Of particular importance, the early stages of esophageal and gastric neoplasms are asymptomatic as well. These patients are considered to have reached the endpoint of the study if they are diagnosed by EGD and biopsy at 6 months post-randomization.

The present study includes two subgroup analyses, one is *H. pylori* infection status and the other is DAPT treatment. The results of our DACAB-GI-1 study [12] showed that the positive rate of *H. pylori* in patients receiving 1 year of antiplatelet therapy after CABG was 38.5%. After the study is completed, patients will be divided into the *H. pylori*-positive group and the *H. pylori*-negative group based on the results of the ^13^C urea breath test before enrollment; in addition, patients will be categorized into the clopidogrel plus aspirin group and the ticagrelor plus aspirin group according to their DAPT regimen after CABG. The difference of the primary and secondary endpoints will be compared in the above two subgroup analyses, respectively. Recently, the OPT-PEACE study [22] revealed the presence and severity of gastrointestinal mucosal lesions at 6 months or 12 months by magnetically controlled capsule endoscopy in patients receiving 12-month DAPT without PPI after PCI. To date, no study has reported visually evaluating the upper gastrointestinal mucosal injury (including RE) after 6 and 12 months of PPI treatment combined with two different DAPT by EGD yet.

In conclusion, the present study (DACAB-GI-2) probes the difference in preventing DAPT-related upper gastrointestinal mucosal injury after CABG between the different durations of pantoprazole treatment. The results should provide some confirmatory evidence for studies in relevant fields.

### Trial status

Recruitment starts on October 20, 2019. A total of 236 patients were enrolled in the DACAB-GI-2 trial between October 2019 and February 2022. Among them, 183 patients who received an effective randomized intervention were included in the study. A 12-month clinical follow-up has been completed in 117 patients. Due to the outbreak of Omicron in March 2022, Shanghai is currently still in a city-wide lockdown. Some follow-up visits have had to be postponed for 2 to 3 months. The duration of the trial was extended with the approval of the ethics committee, and it is planned to be completed in June 2023. The version of this protocol is 4.0 (date 20220225).

## Data Availability

The datasets used and/or analyzed during the current study are available from the corresponding authors on reasonable request.

## Abbreviations

DAPT: Dual antiplatelet therapy
CABG: Coronary artery bypass grafting
PPIs: Proton pump inhibitors
RCT: Randomized controlled trial
EGD: Esophagogastroduodenoscopy
RE: Reflux esophagitis
MACE: Major adverse cardiovascular events
GERD: Gastroesophageal reflux disease
CYP3A4: Cytochrome P450 3A4
NSAIDs: Non-steroidal anti-inflammatory drugs
CRF: Case report form
TC: Telephone calls
AEs: Adverse events
CTA: Computed tomographic angiography
CAG: Coronary angiography
mFAS: Modified full analysis set
PPS: Per-protocol set
SS: Safety analysis set

## References

[1] Kulik A, Ruel M, Jneid H, Ferguson TB, Hiratzka LF, Ikonomidis JS (2015). Secondary prevention after coronary artery bypass graft surgery: a scientific statement from the American Heart Association. Circulation.

[2] Koskinas KC, Räber L, Zanchin T, Wenaweser P, Stortecky S, Moschovitis A, et al. Clinical impact of gastrointestinal bleeding in patients undergoing percutaneous coronary interventions. Circ Cardiovasc Interv. 2015;8. 10.1161/CIRCINTERVENTIONS.114.002053 25910501

[3] Bhatt DL, Scheiman J, Abraham NS, Antman EM, Chan FK, Furberg CD (2008). ACCF/ACG/AHA 2008 expert consensus document on reducing the gastrointestinal risks of antiplatelet therapy and NSAID use: a report of the American College of Cardiology Foundation Task Force on Clinical Expert Consensus Documents. Circulation.

[4] Moukarbel GV, Bhatt DL (2012). Antiplatelet therapy and proton pump inhibition: clinician update. Circulation.

[5] Chinese Expert Consensus Group on the Prevention and Treatment of Digestive Tract Injury with Antiplatelet Drugs. Chinese expert consensus on the prevention and treatment of gastrointestinal injury by antiplatelet drugs (2012 update). Chinese Journal of Internal Medicine. 2013;52:264-270.

[6] Li XQ, Andersson TB, Ahlström M, Weidolf L (2004). Comparison of inhibitory effects of the proton pump-inhibiting drugs omeprazole, esomeprazole, lansoprazole, pantoprazole, and rabeprazole on human cytochrome P450 activities. Drug Metab Dispos.

[7] El Rouby N, Lima JJ, Johnson JA (2018). Proton pump inhibitors: from CYP2C19 pharmacogenetics to precision medicine. Expert Opin Drug Metab Toxicol.

[8] Valgimigli M, Bueno H, Byrne RA, Collet JP, Costa F, Jeppsson A (2018). 2017 ESC focused update on dual antiplatelet therapy in coronary artery disease developed in collaboration with EACTS: the Task Force for dual antiplatelet therapy in coronary artery disease of the European Society of Cardiology (ESC) and of the European Association for Cardio-Thoracic Surgery (EACTS). Eur Heart J.

[9] Levine GN, Bates ER, Bittl JA, Brindis RG, Fihn SD, Fleisher LA (2016). 2016 ACC/AHA guideline focused update on duration of dual antiplatelet therapy in patients with coronary artery disease: a report of the American College of Cardiology/American Heart Association Task Force on Clinical Practice Guidelines. J Am Coll Cardiol.

[10] Wallentin L, Becker RC, Budaj A, Cannon CP, Emanuelsson H, Held C (2009). Ticagrelor versus clopidogrel in patients with acute coronary syndromes. N Engl J Med.

[11] Storey RF, Angiolillo DJ, Patil SB, Desai B, Ecob R, Husted S (2010). Inhibitory effects of ticagrelor compared with clopidogrel on platelet function in patients with acute coronary syndromes: the PLATO (PLATelet inhibition and patient Outcomes) PLATELET substudy. J Am Coll Cardiol.

[12] Tang C, Zhu Y, Yang X, Xu B, Ye C, Yang Y (2020). Upper gastrointestinal mucosal injury associated with ticagrelor plus aspirin, ticagrelor alone, or aspirin alone at 1-year after coronary artery bypass grafting. J Gastroenterol Hepatol.

[13] Nam SJ, Lee HS, Lim YJ (2018). Evaluation of gastric disease with capsule endoscopy. Clin Endosc.

[14] Weledji EP (2020). Acute upper gastrointestinal bleeding: a review. Surg Pract Sci.

[15] Oakland K, Chadwick G, East JE, Guy R, Humphries A, Jairath V (2019). Diagnosis and management of acute lower gastrointestinal bleeding: guidelines from the British Society of Gastroenterology. Gut.

[16] Muthusamy VR, Lightdale JR, Acosta RD, Chandrasekhara V, Chathadi KV, Asge Standards of Practice Committee (2015). The role of endoscopy in the management of GERD. Gastrointest Endosc.

[17] Park J, Cho YK, Kim JH (2018). Current and future use of esophageal capsule endoscopy. Clin Endosc.

[18] Lanza FL, Marathi UK, Anand BS, Lichtenberger LM (2008). Clinical trial: comparison of ibuprofen-phosphatidylcholine and ibuprofen on the gastrointestinal safety and analgesic efficacy in osteoarthritic patients. Aliment Pharmacol Ther.

[19] Fitzgibbon GM, Kafka HP, Leach AJ, Keon WJ, Hooper GD, Burton JR (1996). Coronary bypass graft fate and patient outcome: angiographic follow-up of 5,065 grafts related to survival and reoperation in 1,388 patients during 25 years. J Am Coll Cardiol.

[20] Zhao Q, Zhu Y, Xu Z, Cheng Z, Mei J, Chen X (2018). Effect of ticagrelor plus aspirin, ticagrelor alone, or aspirin alone on saphenous vein graft patency 1 year after coronary artery bypass grafting: a randomized clinical trial. JAMA.

[21] Chan FKL, Goh KL, Reddy N, Fujimoto K, Ho KY, Hokimoto S (2018). Management of patients on antithrombotic agents undergoing emergency and elective endoscopy: joint Asian Pacific Association of Gastroenterology (APAGE) and Asian Pacific Society for Digestive Endoscopy (APSDE) practice guidelines. Gut.

[22] Han Y, Liao Z, Li Y, Zhao X, Ma S, Bao D (2022). Magnetically controlled capsule endoscopy for assessment of antiplatelet therapy-induced gastrointestinal injury. J Am Coll Cardiol.

